# Influencing Factors and Implementation Pathways of Adherence Behavior in Intelligent Personalized Exercise Prescription: Qualitative Study

**DOI:** 10.2196/59610

**Published:** 2024-12-05

**Authors:** Xuejie Xu, Guoli Zhang, Yuxin Xia, Hui Xie, Zenghui Ding, Hongyu Wang, Zuchang Ma, Ting Sun

**Affiliations:** 1 School of Nursing Bengbu Medical University Bengbu China; 2 Institute of Intelligent Machines Hefei Institutes of Physical Sciences Chinese Academy of Sciences Hefei China; 3 Department of Physical Education and Arts Bengbu Medical University Bengbu China

**Keywords:** exercise prescription, adherence behavior, qualitative, influence factors, Transtheoretical Model, multiple motivations of behavior

## Abstract

**Background:**

Personalized intelligent exercise prescriptions have demonstrated significant benefits in increasing physical activity and improving individual health. However, the health benefits of these prescriptions depend on long-term adherence. Therefore, it is essential to analyze the factors influencing adherence to personalized intelligent exercise prescriptions and explore the intrinsic relationship between individual behavioral motivation and adherence. This understanding can help improve adherence and maximize the effectiveness of such prescriptions.

**Objective:**

This study aims to identify the factors influencing adherence behavior among middle-aged and older community residents who have been prescribed personalized exercise regimens through an electronic health promotion system. It also explores how these factors affect the initiation and maintenance of adherence behavior.

**Methods:**

We used purposive sampling to conduct individual, face-to-face semistructured interviews based on the Transtheoretical Model (TTM) with 12 middle-aged and older community residents who had been following personalized exercise regimens for 8 months. These residents had received detailed exercise health education and guidance from staff. The interviews were recorded, transcribed verbatim, and analyzed using NVivo software through grounded theory. We then applied the TTM and multibehavioral motivation theory to analyze the factors influencing adherence. Additionally, the relationship between behavioral motivations and adherence was explored.

**Results:**

Using the behavior change stages of the TTM, open coding yielded 21 initial categories, which were then organized into 8 main categories through axial coding: intrinsic motivation, extrinsic motivation, benefit motivation, pleasure motivation, achievement motivation, perceived barriers, self-regulation, and optimization strategies. Selective coding further condensed these 8 main categories into 3 core categories: “multitheory motivation,” “obstacle factors,” and “solution strategies.” Using the coding results, a 3-level model of factors influencing adherence to intelligent personalized exercise prescriptions was developed. Based on this, an implementation path for promoting adherence to intelligent personalized exercise prescriptions was proposed by integrating the model with the TTM.

**Conclusions:**

Adherence to personalized exercise prescriptions is influenced by both facilitating factors (eg, multibehavioral motivation, optimization strategies) and obstructive factors (eg, perceived barriers). Achieving and maintaining adherence is a gradual process, shaped by a range of motivations and factors. Personalized solutions, long-term support, feedback mechanisms, and social support networks are essential for promoting adherence. Future efforts should focus on enhancing adherence by strengthening multibehavioral motivation, optimizing solutions, and addressing barriers to improve overall adherence.

## Introduction

Mounting evidence suggests that regular moderate physical activity significantly improves health-related quality of life, promotes community engagement and healthy aging, and may enhance cognition and mental health [1,2]. It also appears to mitigate the risk of at least 35 chronic diseases and reduce mortality [1,3-6]. Physical activity is currently recognized as a sustainable approach to promoting individual and community health and well-being [7]. Exercise prescription [8], a structured program designed to guide exercise for health promotion and the prevention and management of chronic diseases, has been applied to diverse populations, including healthy individuals, those with sports injuries, perioperative patients, individuals with chronic conditions, and those with disabilities [9-11]. However, many physicians struggle to provide physical activity guidance and prescribe scientifically effective exercise regimens due to a lack of knowledge, training, awareness, and understanding of exercise rehabilitation [3]. These challenges, combined with the absence of standardized implementation guidelines [12] and other barriers, hinder the promotion and integration of exercise prescriptions.

Most exercise guidelines [13-16] strongly recommend personalized and well-defined exercise prescriptions that specify the mode, intensity, frequency, and duration of exercise. Traditional exercise prescriptions often struggle to achieve scientific personalization, prompting the development of intelligent, personalized alternatives. By incorporating advanced technologies such as artificial intelligence and big data analysis, these modern approaches provide individuals with more precise, scientifically grounded, and customized exercise plans. For example, Netz et al [17,18] developed an innovative tool for remotely assessing balance, strength, and flexibility in middle-aged and older individuals, providing personalized exercise plans via smartphones. Lin et al [19] and Sun et al [20] created and validated distinct systems: a force gauge system that integrates exercise games with the Internet of Things and a cloud-based intelligent personalized exercise prescription system. Both systems are designed to deliver tailored exercise prescriptions for middle-aged and older individuals. Additionally, a multimodal data-driven artificial intelligence system has been developed and validated to generate personalized exercise prescriptions for patients with mental disorders [21]. These intelligent tools and systems effectively address the limitations of conventional exercise prescription methods. Beyond significantly improving exercise outcomes, reducing exercise-related risks, and enhancing individual health and quality of life, they also alleviate the burden caused by the shortage of professional sports rehabilitation resources, thereby improving the quality and accessibility of sports rehabilitation services [19-22]. Most research on intelligent personalized exercise prescriptions has focused on system design and development [23] and efficacy validation [17]. However, studies examining the factors that influence individual adherence behaviors and their underlying pathways are currently lacking. Recent systematic reviews and meta-analyses [24-26] suggest that while smart health interventions hold promise for improving exercise adherence, the specific factors and pathways driving adherence have not been fully explored or systematically explained. This gap makes it challenging for individuals to maintain long-term adherence. Therefore, it is crucial to further investigate and refine the factors, pathways, and theoretical frameworks related to adherence to personalized smart exercise prescriptions.

Motivation is a critical determinant in shaping individuals’ behavioral intentions and driving behavioral changes, with various motivational factors regulating and influencing individual behavior [27-29]. Although motivation significantly impacts adherence behavior, theoretical limitations, a lack of empirical research, and the complexity of individual motivations contribute to a theoretical “black box” regarding how these motivations influence adherence to exercise prescriptions. The relationship between behavioral motivation and individual adherence behavior remains underexplored [30-32]. In health promotion, several theoretical models [33,34] have been used to study individual behavior. Among these, the Transtheoretical Model (TTM) has been widely applied in research on digital health behavior and behavior change [35-37]. By dividing behavior change into 5 stages—precontemplation, contemplation, preparation, action, and maintenance—the TTM provides a detailed framework for understanding the mechanisms and pathways of individual behavior change [38]. The TTM has shown promising results in various social studies focused on health behavior change and health promotion [39-41].

Therefore, this study uses qualitative methods to explore the factors influencing the adherence behavior of community-dwelling middle-aged and older residents who have followed personalized exercise prescriptions issued through an electronic health promotion system for 8 months. By integrating the TTM, the study analyzes behavioral intentions and changes at different stages of behavior change, identifies key factors in the adherence behavior change process, and constructs an implementation path model for adherence to intelligent personalized exercise prescriptions, driven by behavioral motivations and other influencing factors. This study reveals how behavioral motivations drive and sustain adherence to exercise prescriptions, expands the application of the TTM in digital health services, and provides insights for future research on adherence to intelligent personalized exercise prescriptions. Additionally, it offers practical strategies for enhancing and maintaining long-term adherence to these prescriptions. To our knowledge, this is the first qualitative study on adherence behavior to intelligent personalized exercise prescriptions.

## Methods

### Design

This study is part of a longitudinal research project aimed at examining the health impacts of intelligent personalized exercise prescriptions on community-dwelling middle-aged and older residents. It uses a descriptive exploratory qualitative design, based on face-to-face semistructured interviews. The study strictly followed the COREQ (Consolidated Criteria for Reporting Qualitative Research) guidelines [42].

### Participants

The participants in this study are a subsample from a longitudinal research project conducted by the research team, which aims to investigate the long-term effects of intelligent personalized exercise prescriptions on the health of middle-aged and older individuals. The inclusion criteria for the longitudinal study were age 50 years or older; no severe physical diseases or related complications; no cognitive or mental disorders among community-dwelling middle-aged and older residents; and exclusion of individuals with severe cardiovascular, pulmonary, or renal diseases, severe diabetes or related complications, fasting blood glucose of 13.3 mmol/L or higher with positive urine ketones, postprandial blood glucose of 19.4 mmol/L or higher, resting blood pressure of 180/110 mmHg or higher, or severe cognitive or mental disorders. Participants were initially recruited from the community via telephone and verbal invitations.

To investigate adherence behavior and the factors influencing adherence to intelligent personalized exercise prescriptions, we used purposive sampling to select participants for face-to-face semistructured interviews. The inclusion criteria were (1) community-dwelling middle-aged and older residents who had undergone home-based health checkups between 2021 and 2022 and received personalized exercise prescriptions through an electronic health promotion system administered by community staff; (2) participants who were provided with printed exercise materials and received detailed explanations and guidance from community staff; and (3) individuals whose exercise prescriptions had been active for at least 8 months. To gain a comprehensive understanding of exercise prescription adherence, we specifically recruited participants with neutral or negative attitudes toward intelligent personalized exercise prescriptions, as well as those who self-reported low adherence. The sample size was determined based on theoretical saturation, defined as the point at which no new issues or insights emerge and all relevant conceptual categories have been identified and explored [43]. Throughout the research process, the team continuously analyzed the dialogues and assessed the saturation level of the interview data at each stage. After the 12th interview, no new information emerged, all major concepts and categories had been thoroughly identified and explored, and theoretical saturation was reached. As a result, 12 eligible community-dwelling middle-aged and older residents participated in the face-to-face semistructured interviews.

### Ethics Considerations

This study received ethical approval from the Human Research Ethics Committee of Bengbu Medical University (approval number 2022-103).

### Intelligent Personalized Exercise Prescription Program

Details of the intelligent personalized exercise prescription system have been previously described [20]. The system generally consists of 4 components: user registration and information input, internet-based health monitoring devices, online questionnaire surveys, and a cloud platform.

Before generating a personalized exercise prescription, participants must register with their real names and provide personal information, including basic details (age, gender, height, and weight) and health history (past medical history and exercise habits). Next, participants’ health data are collected using internet-based health monitoring devices. Additionally, participants must complete a series of online health questionnaires to provide the necessary data for generating personalized exercise prescriptions. Finally, all collected health data are uploaded to a cloud platform. The platform uses advanced data analysis techniques and artificial intelligence algorithms to evaluate participants’ health data and generate personalized exercise prescriptions tailored to each individual.

Once the personalized exercise prescription is generated, community health service staff will provide face-to-face health education and guidance based on it. Depending on participants’ needs, the prescription can be delivered in either electronic or paper form, promoting better adherence.

**Figure 1:**
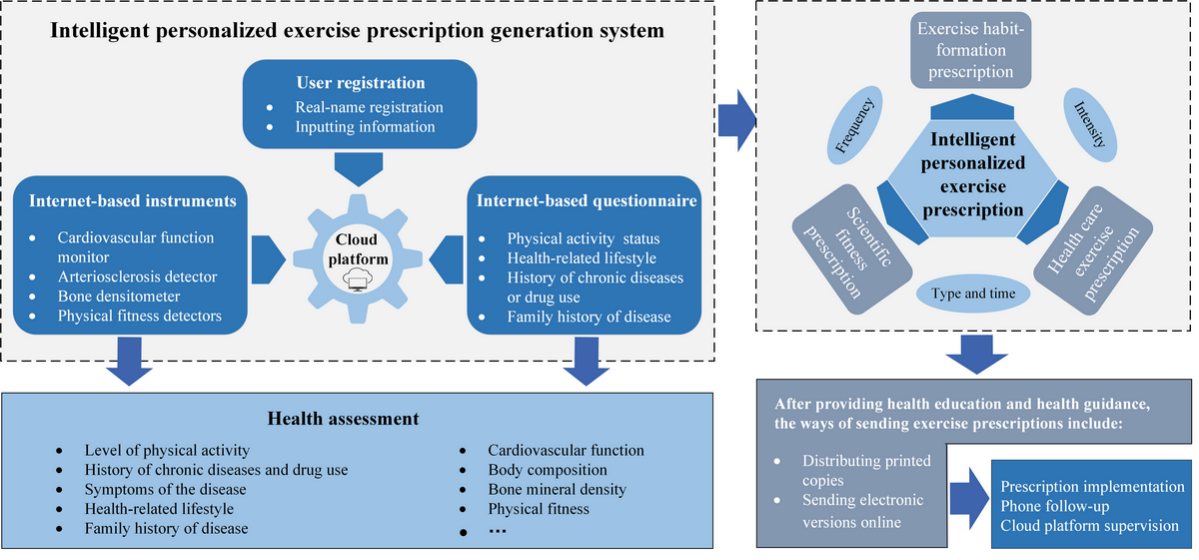
Generation and application of intelligent personalized exercise prescription.

After the exercise prescription is implemented, community health service staff can access user information via the cloud platform and conduct regular follow-up calls. These calls aim to remind participants to adhere to the exercise prescription, answer their questions, and provide necessary support and guidance. Figure 1 illustrates the structure of the intelligent personalized exercise prescription system and the process of generating and applying the prescriptions.

### Procedures and Data Collection

This study strictly adheres to the COREQ [42]. To gather detailed data and gain a deeper understanding of participants’ subjective experiences and true feelings, semistructured interviews were conducted. All interviews were carried out by 3 trained researchers, including the first author (XX), a graduate nursing student, and an undergraduate nursing student, between February and March 2023. Following purposive sampling to select interviewees, rapport was established between the interviewers and participants. Interviews were conducted in quiet, private spaces to minimize interruptions. Before the interviews, participants were briefed on the research objectives, methods, expected duration, and confidentiality principles. Informed consent was obtained, and participants signed consent forms. The interviews were audio-recorded by the interviewers, with additional on-site notes taken for further analysis. Additionally, interviewers observed and promptly documented participants’ nonverbal behaviors, including tone, gestures, and facial expressions, during the interviews. Each interview lasted 20-30 minutes. Within 24 hours of completion, the recordings were transcribed into written transcripts and verified by the participants for content accuracy.

According to the main principles of the TTM, the occurrence and maintenance of individual behavior change occur across multiple stages. Therefore, before conducting the semistructured interviews, we determined the landmark events for each stage—“intention,” “preparation,” “action,” and “maintenance”—based on the research objectives and the definitions and characteristics of these stages in the TTM. Relevant questions about stage transitions were formulated in the interview outline to ensure the interviews were systematic and scientifically rigorous. The interview outline included questions about the respondents’ basic information; their perceptions and attitudes toward the intelligent personalized exercise prescription; factors that promote or hinder progression through specific stages of behavior change; and their activities, emotional states, needs, and suggestions within each stage. To ensure confidentiality, all participant information and data were anonymized and deidentified to prevent personal identification. Recordings and transcripts were stored in password-protected, secure files on the university server. The interviews concluded when no new information emerged.

### Analysis

After the qualitative interviews were completed, the initial transcripts of the interview recordings—aligned with the interview questions corresponding to the 4 behavior change stages (“intention stage,” “preparation stage,” “action stage,” and “maintenance stage”) in the TTM—were imported into the qualitative analysis software NVivo 12 (Lumivero). The transcripts were then subjected to a 3-level coding process (Textbox 1) based on grounded theory [44,45] within the framework of the 4 behavior change stages.

Drawing on the TTM and the coding results, we analyzed the factors influencing the adherence behavior to intelligent personalized exercise prescriptions, proposed hypotheses for the pathways to achieving adherence, and developed an implementation path model for adherence to intelligent personalized exercise prescriptions. We validated the hypotheses through follow-up interviews with the 12 respondents and refined the theoretical model based on team discussions, ultimately finalizing it.

For clearer presentation and data referencing, the 12 participants were labeled P1 through P12. Data analysis and organization were primarily carried out by 5 researchers; 3 researchers (XX, GZ, and YX) sorted and classified the interview recordings for open, axial, and selective coding, while the remaining 2 (TS and HX) verified the phased collation and ensured accuracy and consistency in the 3-level coding process. The hypotheses and model framework for adherence behavior pathways to intelligent personalized exercise prescriptions were formulated by the research team, validated through follow-up interviews conducted by 2 researchers (XX and YX), and refined through group discussions.

**The 3-level coding process.**

**1. Open coding**

This is the most basic level of abstraction in the coding process. Each interview transcript was read thoroughly, and the text was coded as it appeared, without unnecessary abstraction. By analyzing the text line by line, we identified and marked concepts and phenomena relevant to the research questions, ensuring that the coding remained original and authentic.

**2. Axial coding**

Building on open coding, we focused on the logical connections and relationships between the identified concepts. Through ongoing comparison and analysis, we refined these into higher-level main categories.

**3. Selective coding**

We further examined the relationships between the main categories, identifying a core category that served as the central focus of the coding system. This core category guided the analysis and discussion of the other categories.

## Results

### Overview

**Figure 2:**
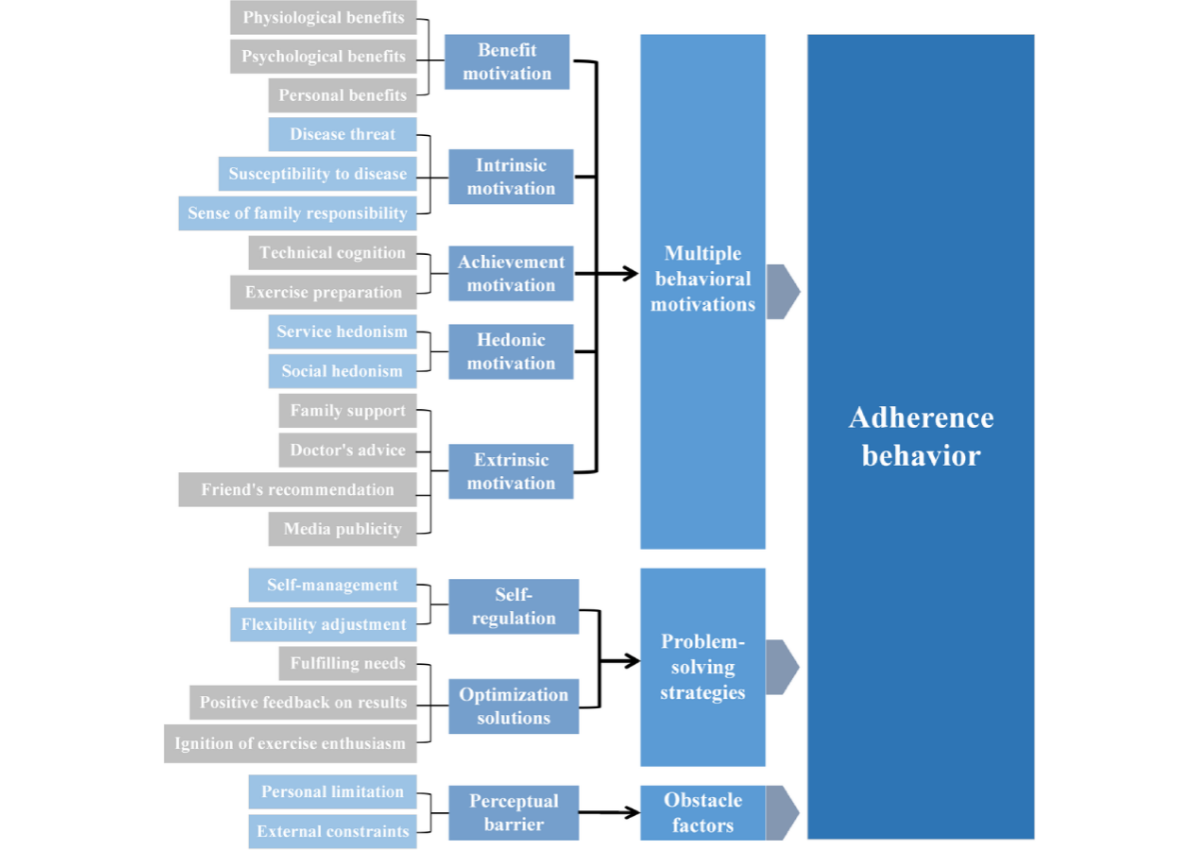
The 3-level model of factors influencing the adherence behavior to intelligent personalized exercise prescription.

Effective interviews were conducted with 12 participants, and their general information is summarized in Table 1. The results of the 3-level coding process based on the 4 stages of behavior change in the TTM are as follows: (1) Open coding involves breaking down, reorganizing, and conceptualizing the transcribed interview materials. This process led to the identification of 21 categories (see Table 2), which highlight the direct factors influencing adherence to intelligent personalized exercise prescriptions. (2) In the axial coding stage, we examined the relationships and logical connections among the 21 categories identified during open coding. This led to the formation of 8 main categories (Table 3). (3) During selective coding, we integrated and refined the existing categories to extract a “core category.” The categories of “hedonic motivation,” “benefit motivation,” “intrinsic motivation,” “extrinsic motivation,” and “achievement motivation” were consolidated into a single category, “multiple motivations of behavior.” Similarly, “self-regulation” and “optimization solutions” were grouped under “problem-solving strategies,” while “perceptual barriers” were categorized as “obstacle factors” (see Table 4). The open coding derived from the original interview data represents direct experiences and situational factors, forming the first level (bottom level). The 21 categories identified during open coding were organized into axial coding, which represents the preliminary classification of motivation and obstacle factors, making it the second level (middle level). Selective coding, which represents the core influencing factors derived from the systematic analysis of the main categories, forms the third level (top level). In summary, we constructed a 3-level model of factors influencing the adherence behavior to intelligent personalized exercise prescriptions, as illustrated in Figure 2.

Based on the TTM, Multiple Behavioral Motivation Theory, and the 3-level coding results from interviews on the 4 behavior change stages, we propose the following hypotheses for the path to adherence to intelligent personalized exercise prescriptions:

- Hypothesis 1: An individual’s intrinsic motivations (such as disease susceptibility, perceived threat, and a sense of family responsibility) and extrinsic motivations (including family support, doctor’s advice, recommendations from friends, and media publicity) contribute to the intention to adhere, leading to the “adherence intention stage.”
- Hypothesis 2: Achievement motivation encourages the individual to reach the “adherence preparation stage,” where they prepare for the actual adherence behavior to the exercise prescription.
- Hypothesis 3: Hedonic and benefit motivations drive the individual to enter the “adherence behavior stage,” resulting in the initiation of actual adherence behaviors.
- Hypothesis 4: The presence of multiple motivations, coupled with problem-solving strategies, leads the individual to the “adherence maintenance stage,” where they progress from partial to full adherence, achieving complete and sustained adherence to the exercise prescription.

**Figure 3:**
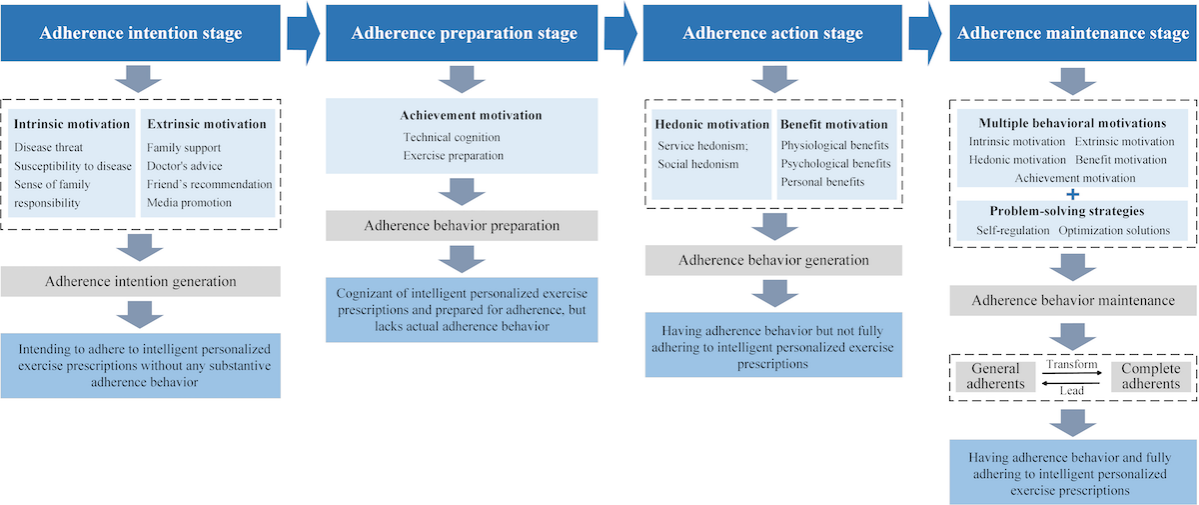
The implementation path model of adherence behavior to intelligent personalized exercise prescription based on the Transtheoretical Model and Multiple Behavioral Motivation Theory.

The path hypotheses were verified and validated by 2 researchers (XX and YX) through follow-up interviews with the 12 respondents. Based on these findings, a model for the realization path of adherence behavior to intelligent personalized exercise prescriptions was further developed. This model consists of 4 stages of adherence: intention, preparation, action, and maintenance. In the intention stage, individuals are motivated by intrinsic and extrinsic factors, developing the intention to adhere, but without engaging in actual behavior. Driven by achievement motivation, they progress to the preparation stage, where they ready themselves for action and set goal expectations, though behavior has yet to occur. During the action stage, hedonic and benefit motivations play a key role in prompting individuals to take action and experience benefits, though full adherence may not yet be achieved. Finally, in the maintenance stage, supported by multiple motivations and problem-solving strategies, individuals transition from general adherents to complete adherents, fully adhering to the exercise prescription, as illustrated in Figure 3.

**Table 1:** General information on interviewees.

Number	Age (years)	Occupation	Gender	Educational level	Presence of chronic diseases	Type of exercise prescription	Previous exercise habits	Previous success or failure in health behavior change (eg, smoking cessation or weight loss)
P1	66	Shop assistant	Female	Junior high school	Yes	Scientific fitness mode	Yes	No
P2	71	Public official	Female	Junior high school	Yes	Scientific fitness mode	Yes	Weight loss success
P3	68	Teacher	Female	Vocational school	No	Health care mode	No	No
P4	72	Laborer	Male	Primary school	Yes	Scientific fitness mode	Yes	No
P5	63	Laborer	Female	Primary school	No	Health care mode	No	No
P6	68	Staff	Male	High school	No	Exercise habit–formation mode	No	Smoking and alcohol cessation success
P7	76	Staff	Male	Junior high school	No	Exercise habit–formation mode	No	No
P8	57	Pharmacy clerk	Female	High school	Yes	Scientific fitness mode	Yes	No
P9	60	Staff	Female	Junior high school	No	Exercise habit–formation mode	No	No
P10	69	Staff	Male	Junior high school	No	Health care mode	No	No
P11	72	Laborer	Female	High school	Yes	Scientific fitness mode	Yes	No
P12	68	Laborer	Male	High school	No	Health care mode	Yes	No

**Table 2:** Categories formed by open coding of interview content.

Behavior change stage, category, and initial concept			Representative original sentences
**Intention stage**			
	**Disease threat**		
		Encountering problems	*My cervical vertebra has some issues. Looking at my phone too much, due to my age, so I exercise to relieve the discomfort in my cervical vertebra.* [P5]
		Worsening of symptoms	*My heart wasn’t very good before. The doctor told me to try to increase the vitality of my heart. The lazier you are, the worse your heart condition gets.* [P7]
	**Susceptibility to disease**		
		Frailty due to aging	*As I’ve gotten older, my health has declined.* [P8]
		Decline in function	*Now our physical functions are not as good as those of younger people. If we don't exercise more, it won’t work.* [P8]
		Hazards of prolonged sitting	*If you don’t exercise and just lie down for a long time without moving, you'll definitely have health problems later on.* [P4]
	**Sense of family responsibility**		
		Reducing children’s worries	*Although my children are grown up, I am still their support. If I am healthy, they will worry less.* [P9]
		Promoting family health	*If I learn scientific exercise methods, I want to exercise with my family. Won’t our family all be healthy then?* [P10]
	**Family support**		
		Support from children	*My children also strongly support me participating in this.* [P5]
		Companionship of spouse	*Usually, my spouse and I play badminton together.* [P1]
	**Doctor’s advice**		
		Doctor’s notification	*The doctor told me to try to increase the vitality of my heart.* [P7]
	**Friend’s recommendation**		
		Recommending participation	My friend Yue ** joined first and spoke highly of your place, so she recommended me to join personalized exercises here. [P6]
	**Media publicity**		
		Broadcasting publicity	*Initially, it was mainly through some radio broadcasts that I learned about the benefits of exercise.* [P5]
**Preparation stage**			
	**Technological awareness**		
		Scientifically expectations	*Your personalized exercise program must be well-organized and scientifically based.* [P1]
		Organizing positive	*This exercise prescription must have benefits for the exercise of a specific body structure or physical fitness.* [P1]
		Authoritative projects	*According to my daughter-in-law, she said, “Mom, there is a health program here in collaboration with a medical college and the University of Science and Technology of China. They provide free health checkups and tailor personalized exercise prescription for you. They also guide how to exercise. They are here to promote health. You should go and take a look. It's much better than those deceptive health products.”* [P2]
	**Exercise preparation**		
		Preparing sports equipment	*In order to adhere to the exercise prescription properly, I even prepared specific exercise clothing and shoes.* [P5]
		Peer support	*At the beginning, I arranged to exercise together with a few people.* [P1]
**Action stage**			
	**Service hedonism**		
		Problem-solving	*It’s great that you can explain any questions we don’t understand so well.* [P3]
		Thorough guidance	*You health providers are so thoughtful in explaining the examination reports and guiding us in exercise.* [P5]
		Careful inspection	*Your staff are very meticulous in every examination, both before and after exercise.* [P4]
		Professionalism of the service provider	*You have a lot of knowledge and explain things very well...* [P12]
		Comprehensive reporting	*The reports you provide are very clear and comprehensive.* [P8]
	**Social hedonism**		
		Making friends	*During exercise, you can also make many friends (especially outdoors).* [P6]
	**Physiological benefits**		
		Symptom control	*Before, I had high blood sugar, but now my blood sugar is also very good.* [P6] *I used to have constipation, and it’s still there, but the frequency has decreased...* [P5]
		Physical improvement	*There has been an improvement in my body. I used to have high blood pressure and unstable blood pressure, but now it's very stable.* [P2]
		Increasing physical activity	*Look, I can raise my arms very high now (demonstrating a posture).* [P2]
	**Psychological benefits**		
		Feeling relaxed	*After exercising, I feel my mood is more relaxed, my body feels better, and I have more energy than before.* [P3]
		Energetic	*Now, both mentally and energetically, I am better than before.* [P2]
		Experiencing well-being	*(After being guided on walking posture) When the heels touch the ground, I involuntarily straighten up, walking with chest out and stomach in, it feels particularly good, this kind of feeling...* [P6]
	**Personal benefits**		
		Acquiring knowledge	*Compared to before, I have learned how to exercise with proper posture, how to relax after exercising, and how to stretch...* [P2]
		Free medical checkups	*They also conducted physical examinations for us, checking our bones and bone density, teaching us how to improve. They told me that my bone density had reached the bottom line and couldn't decline further, advising us to drink milk and eat eggs daily, as well as recommending specific vegetables. They explained it very thoroughly.* [P4]
**Maintenance stage**			
	**Self-management**		
		Perseverance	*I can persevere because that’s my personality. Once I set my mind on something, I persist until I succeed, no matter what obstacles I face.* [P6]
		Forming habits	*To develop a good exercise habit, I have to wake up at a certain time every day.* [P2]
		Strict self-discipline	*I must be strict with myself and not make excuses like “it’s cold today” or “it’s windy” to forgive myself. I need to ensure that I accomplish what needs to be done.* [P2]
	**Flexibility adjustment**		
		Persisting to completion	*If I don’t meet the planned exercise volume today, I’ll make up for it tomorrow by sticking to the previous day’s shortfall.* [P6]
		Adapting flexibly	*I follow the exercise routine as advised by you. On rainy days, I use the treadmill at home, ensuring I meet the required exercise volume while exercising in a way I enjoy.* [P4]
	**Fulfillment of needs**		
		Adding videos	*It would be great if it could be turned into a video. The report is easy to misplace, and it’s easier not to forget with a video, especially at our age...*[P1]
		Enriching images	*I have a small suggestion. There are too few exercise images in our medical reports. It would be nice if they could be more varied. Otherwise, everything else is quite good.* [P10]
	**Positive feedback on results**		
		Good results upon retesting	*After exercising and reassessing my health, I found that I’m doing well. My grip strength is still good, and I’m almost reaching eighty kilograms. I’m still going strong. Hehehe (laughs happily).* [P4]
	**Ignition of exercise enthusiasm**		
		Eagerness to exercise	*After exercising for a while, I feel very enthusiastic about it, as if I’m eager to participate in sports from the bottom of my heart.* [P5]
		Exercising voluntarily	*Having a workout plan makes me feel psychologically engaged. When it’s time, I feel like I should exercise, and there’s a sense of awareness, a kind of concern, that makes me want to work out.* [P6]
**Pan-stage factors**			
	**Personal limitations**		
		Struggling to persist	*Sometimes, doing exercise alone is difficult, so doing group activities helps.* [P7]
		Lack of exercise interest	*However, later on, my legs felt better. Originally, I didn’t like exercising and didn’t do much exercise.* [P3]
		Physical discomfort	*Because of my bad back, my legs hurt when I walk. So, I’m not good at walking and don’t exercise much.* [P3]
		Bodily fatigue	*Sometimes when I feel tired, I don’t feel like exercising.* [P1]
		Prone to forgetfulness	*I can’t remember the exercise prescription because I’m old and forgetful. I used to stick to it at the beginning, but later, I did less, and I always forget the movements.* [P8]
	**External constraints**		
		Lack of resources	*There are no good places near my home. There is a small Friendship Square, but there is no track or facilities.* [P2]
		Unable to free oneself	*The children need to come here for meals, buy groceries in the morning, and cook. It’s usually fine, but sometimes it’s overwhelming when I’m busy.* [P3]

**Table 3:** Main categories formed by interview content axial coding.

Behavior change stage, main category, and category			Definition
**Intention stage**			
	**Intrinsic motivation**		
		Disease threat	Perception of adverse health consequences resulting from one’s current condition.
		Susceptibility to disease	Adverse physical conditions, declining quality, and unhealthy lifestyle habits are more likely to trigger diseases.
		Sense of family responsibility	Reduce the burden on the family and family members’ worries and promote the health, harmony, and stable development of the family.
	**Extrinsic motivation**		
		Family support	Material or emotional support provided by family members.
		Doctor’s advice	Reasonable advice from doctors based on individual conditions to improve individual situations.
		Friend’s recommendation	Verbal introductions from friends that are hoped to be accepted and adopted.
		Media publicity	Dissemination and transmission of information through information dissemination tools.
**Preparation stage**			
	**Achievement motivation**		
		Technological awareness	Understanding and familiarizing oneself with the intelligent personalized exercise prescription system.
		Exercise preparation	Planning and preparing for upcoming exercise activities.
**Action stage**			
	**Hedonic motivation**		
		Service hedonism	Enjoy and experience high-quality, professional exercise testing and guidance services.
		Social hedonism	Expand social connections, make friends, and enjoy the pleasure and benefits of social interaction.
	**Benefit motivation**		
		Physiological benefits	Control of adverse physiological symptoms of diseases and improvement in physical function or capability.
		Psychological benefits	Improved mental state and psychological well-being, as well as increased energy.
		Personal benefits	Personal access to free benefits, such as exercise and health knowledge, physical fitness, and health assessments.
**Maintenance stage**			
	**Self-regulation**		
		Self-management	Self-control and using inner strength for behavior change or habit formation.
		Flexibility adjustment	Flexibly adjust according to individual circumstances within a certain range.
	**Optimization solutions**		
		Fulfilling needs	Fulfill the requirements arising from individual needs.
		Positive feedback on results	Following the positive feedback from intelligent personalized exercise prescription to encourage better adherence.
		Ignition of exercise enthusiasm	Stimulating or enhancing interest and enthusiasm for exercise through various means.
**Pan-stage factors**			
	**Perceptual barriers**		
		Personal limitation	Limitations imposed by individual conditions on accomplishing tasks.
		External constraints	Limitations imposed by external factors beyond the individual on accomplishing tasks.

**Table 4:** Core categories formed by interview content selective coding.

Core category and main category		Definition
**Multiple behavioral motivations**		
	Hedonic motivation	Behavioral motivation generated from the pursuit of the pleasant feelings brought by exercise.
	Benefit motivation	Behavioral motivation arising from the actual benefits that can be obtained by following the exercise prescription.
	Intrinsic motivation	Behavioral motivation originating from the individual’s internal needs and perceptions.
	Extrinsic motivation	Behavioral motivation resulting from the support and incentives of external factors.
	Achievement motivation	Behavioral motivation arising from the expectation of achieving good performance or reaching specific goals by adhering to the exercise prescription.
**Problem-solving strategies**		
	Self-regulation	Self-management and adjustment of an individual’s behavior or thinking for better compliance with the exercise prescription.
	Optimization solutions	Improvement measures and solutions for enhancing the compliance effect and adherence to the exercise prescription.
**Obstacle factors**		
	Perceptual barriers	Difficulties and obstacles that an individual subjectively perceives as restricting their exercise in accordance with the exercise prescription.

### Factors Influencing Adherence Behavior With Intelligent Personalized Exercise Prescription and Pathways for Implementation

#### Multiple Behavioral Motivational Factors

Motivation is the central factor driving behavior change and serves as the intrinsic force behind individuals’ actions [46,47]. This study explores how various motivations—hedonic, benefit, intrinsic, extrinsic, and achievement—affect participants’ adherence to intelligent personalized exercise prescriptions. These motivations directly influence adherence behaviors [48]. (1) Intrinsic motivations (eg, perceived disease threat and susceptibility, sense of family responsibility) and extrinsic motivations (eg, family support, doctor recommendations, friend referrals, and media publicity) drive participants to develop an intention to adhere to the intelligent personalized exercise prescription. This phase, referred to as the “adherence intention stage,” involves participants forming the intention to adhere, but without yet engaging in actual adherence behaviors. (2) After forming the intention to adhere, participants are driven by achievement motivation, aiming to reach self-set goals through adherence to the exercise prescription. At this stage, they begin preparing for adherence by seeking information about the exercise prescription and acquiring the necessary equipment. This phase, known as the “adherence preparation stage,” builds upon the previous stage but does not yet involve actual adherence behavior. (3) When participants have a strong intention to adhere and are well-prepared, they enter the “adherence action stage” [49], where actual adherence behavior takes place. During this stage, high-quality health guidance, positive service experiences, and expanded social connections foster hedonic motivation. Additionally, the physiological and psychological benefits of adherence, increased health knowledge, and free health checkups provided by health care providers contribute to benefit motivation. Furthermore, hedonic and benefit motivations play a key role in sustaining and further developing adherence behavior during this phase. Therefore, the various motivations influencing participants in the context of intelligent personalized exercise prescriptions are crucial in the first 3 stages and serve as the primary drivers of adherence behavior.

#### Obstacles

“Perceptual barriers,” or obstacles, are critical factors affecting adherence behavior. These barriers can undermine an individual’s confidence and motivation, thereby hindering the sustainability of adherence. They negatively impact both the initiation and maintenance of adherence [50]. The findings by Kilgour et al [51] suggest that these obstacles stem from both individual limitations and external constraints. Individual limitations include disliking exercise, difficulty adhering due to personal traits, memory decline that causes the exercise prescription to be forgotten, and physical ailments or fatigue that prevent adherence. External constraints include insufficient exercise facilities, lack of necessary equipment, and a busy family life that interferes with following the exercise prescription. These factors impede the sustainability of adherence behavior and may lead participants to discontinue or abandon it. As obstacle factors do not occur exclusively in a specific stage of adherence behavior transformation but rather persist throughout each stage, they are classified as “pan-stage factors” during the coding process and the division of behavior change stages. These factors are independent of the 4 distinct stages of behavior change.

#### Solution Strategies

From the qualitative interview data, we extracted shared experiences, suggestions, and needs from participants, which were ultimately distilled into another key factor influencing adherence behavior: “problem-solving strategies.” These strategies help promote the initiation and continuity of adherence behavior. “Problem-solving strategies” encompass participants’ self-regulation and the optimization and enhancement of the exercise prescription intervention schemes. Participants can better promote adherence behavior through self-management, strict self-discipline, habit formation, resilience enhancement, and flexible adjustment of exercise time or location based on individual circumstances. Health service providers and intervention planners can address participants’ needs by incorporating relevant images and videos into the exercise prescriptions, thereby enhancing participants’ motivation and enthusiasm for exercise. They can also regularly schedule or appropriately increase the frequency of health checkups, allowing participants to experience the positive health outcomes of adhering to the exercise prescription. This, in turn, generates positive feedback and promotes the sustained continuity of adherence behavior.

With diverse motivations and problem-solving strategies, adherence behavior progresses into the “adherence maintenance stage.” In this phase, individuals who initially adhered gradually transition to full adherence. Fully adherent individuals then become role models, leading and motivating others to achieve complete adherence.

## Discussion

### Principal Findings

Overall, this study explores adherence behavior to intelligent personalized exercise prescriptions, identifies key influencing factors, and constructs a model that outlines the adherence pathway. The findings indicate that behavioral motivation is the primary driver of adherence, with motivations varying across stages. Intrinsic and extrinsic motivations predominantly influence the early stages, while benefit and hedonic motivations become more significant during the maintenance stage. This suggests that intervention strategies should be dynamically tailored to the individual’s stage of behavior change. Additionally, self-regulation and optimization strategies play a crucial role in promoting adherence. Perceived barriers, such as individual limitations and external conditions, can hinder adherence. Moreover, diverse behavioral motivations drive the entire adherence process, facilitating the transition from intention to preparation, action, and ultimately to maintenance.

### Enhancing Intrinsic and Extrinsic Motivations to Promote Adherence Intentions and Behavior Preparation

Consistent with the findings reported by Almagro et al [52], both intrinsic and extrinsic motivations contribute to the formation of adherence intentions, signaling the beginning of the intention stage. During this stage, individuals are motivated by intrinsic factors, such as perceived disease threat and susceptibility, as well as extrinsic factors, including support and encouragement from family and society, to adhere to intelligent personalized exercise prescriptions. However, individuals have not yet taken concrete action at this stage. To facilitate progression, it is essential to enhance health education and improve health literacy, thereby strengthening intrinsic motivation. Additionally, providing diverse forms of social support (eg, from family, friends, and doctors) can bolster extrinsic motivation [34,35]. Once individuals transition into the preparation stage, they develop a clearer understanding of their adherence goals and begin making preparations. At this stage, achievement motivation becomes the dominant driving force behind behavioral change.

### Satisfying Individual Motivations and Aligning Feedback Mechanisms to Enhance the Maintenance of Adherence Behavior

When individuals begin to engage in adherence behavior, they enter the action stage. At this point, their hedonic and benefit motivations are partially fulfilled, which reinforces their adherence. However, full adherence may not yet be realized. Positive feedback from the outcomes of their behavior plays a critical role in sustaining adherence [53,54]. Therefore, it is essential to provide timely and encouraging feedback during this stage to support continued adherence. A positive feedback loop can enhance patients’ exercise self-efficacy, thereby promoting sustained adherence. When individuals’ diverse motivations are fulfilled and their adherence challenges are addressed through effective strategies, they transition fully from the action stage to the maintenance stage. In this stage, partial adherents gradually evolve into full adherents. These fully adherent individuals, in turn, can serve as role models, guiding and supporting those still in the action stage, helping them achieve full adherence.

### Application of the TTM in Constructing Pathways for the Realization of Adherence Behavior

The precontemplation stage of the TTM refers to individuals who have no intention of changing their behavior within the next 6 months and are not considering any changes. This study, which uses a descriptive exploratory qualitative design within a longitudinal investigation of the effects of intelligent personalized exercise prescriptions on the health of middle-aged and older community residents, includes only participants who have already expressed an intention to change their behavior. Therefore, participants in the precontemplation stage are excluded from this study. As a result, the adherence pathway model developed for this research includes only the 4 stages of the TTM: intention, preparation, action, and maintenance. Additionally, integrating the Multiple Behavioral Motivation Theory with the TTM provides a comprehensive framework for understanding and enhancing adherence to exercise prescriptions. Future research should explore the interactions among various motivational factors and examine their specific effects on adherence behavior.

### Enhancing Individual Self-Efficacy to Promote Effective Adherence Behavior

Through qualitative interviews and subsequent analysis, we found that participants with prior successful behavior change experiences (eg, quitting smoking, reducing alcohol consumption, or losing weight) exhibited a stronger intention to adhere to intelligent personalized exercise prescriptions. They also demonstrated better and more sustained adherence compared with others. This can be attributed to their previous successes, which enhanced their self-efficacy for behavior change [50,55,56]. Therefore, future interventions aimed at improving adherence should focus on boosting individual self-efficacy to facilitate effective and lasting adherence behaviors.

### Strategies for Promoting, Implementing, and Optimizing Personalized Smart Exercise Prescription in the Community

To enhance the promotion, implementation, and optimization of personalized smart exercise prescriptions, we propose several strategies. First, targeted exercise health education and training activities should be organized within the community to improve exercise literacy and adherence intentions among middle-aged and older residents. Additionally, utilizing channels such as social media and community bulletin boards to disseminate information can help increase awareness and acceptance of personalized smart exercise prescriptions. Second, social cognitive methods have proven effective in promoting and maintaining health behavior changes [57]. Thus, using diverse social support strategies can enhance and sustain adherence to personalized smart exercise prescriptions. For example, creating a support network that includes family, friends, and health care providers can offer emotional support, encouragement, and supervision, helping individuals overcome challenges during exercise.

Additionally, establishing regular feedback mechanisms and offering appropriate incentives are crucial for enhancing adherence behavior [58]. Future efforts should consider utilizing smart health devices or apps to provide timely feedback on exercise performance and health improvements for individuals following personalized smart exercise prescriptions [59,60]. Based on this feedback, the exercise prescription can be continuously optimized and adjusted. For example, individuals without access to exercise spaces could be provided with home-based exercise plans. Additionally, adjusting the intensity, frequency, and type of exercises within the prescription can help overcome obstacles, enhance exercise achievements, and improve adherence to the personalized smart exercise prescription. Regarding incentives, implementing reward systems such as point-based rewards and fitness competitions [61] could stimulate both intrinsic and extrinsic motivations, thus promoting and maintaining adherence behavior. Finally, establishing a long-term tracking mechanism to regularly assess adherence to the personalized smart exercise prescription and overall health status is essential. Providing ongoing support and guidance is crucial for helping individuals maintain adherence behavior over the long term.

### Study Limitations

First, this exploratory study has a limited sample size. Although small, the sample has reached theoretical saturation, meaning that adding more participants is unlikely to yield new insights. Second, despite using purposive sampling, selection bias may still be present, as participants with positive attitudes or better adherence to the personalized smart exercise prescription may have been more inclined to participate. To mitigate this, we specifically recruited participants with moderate to low attitudes toward personalized smart exercise prescriptions or those who self-reported low adherence. Additionally, the study focused exclusively on middle-aged and older community residents, excluding other potentially relevant groups, such as working adults or adolescents. This limitation affects the generalizability of the findings. Therefore, future research should include a broader range of populations for comparative analysis to provide a more comprehensive understanding of adherence behavior and its influencing factors across different groups.

Furthermore, qualitative research methods are inherently subjective. Although our study used rigorous qualitative analysis to minimize bias, incorporating quantitative methods could provide more objective data, thereby enhancing the credibility and scientific rigor of the results. Future research should adopt a mixed methods approach, combining both qualitative and quantitative data to gain a more comprehensive understanding. This study, as a qualitative component of a longitudinal investigation on the effects of intelligent personalized exercise prescriptions for middle-aged and older community residents, focuses on a specific cloud-based system. As a result, the generalizability of the findings may be limited. Future research should explore different intelligent personalized exercise prescription systems to validate the broader applicability of the findings. Additionally, the relatively low educational level of the participants may affect the representativeness of the results. Future studies should include participants with diverse educational backgrounds to ensure a more varied sample, gather comprehensive data, and enhance the representativeness of the findings.

Finally, the short duration of this study limited our ability to track long-term adherence behavior. Future research should incorporate long-term follow-ups to assess sustained changes in adherence and provide more comprehensive insights and practical guidance.

### Conclusions

This study adopts a descriptive, exploratory, qualitative design nested within a longitudinal study examining the impact of intelligent personalized exercise prescriptions on the health of middle-aged and older community residents. Through individual, face-to-face, semistructured interviews with 12 participants who had adhered to the intelligent personalized exercise prescription for at least 8 months, received exercise health education materials, and received detailed explanations and guidance from staff, we identified 8 main categories and 3 core categories, integrating the TTM and various behavioral motivations. Furthermore, we developed a model illustrating the factors influencing adherence to intelligent personalized exercise prescriptions and the pathway of adherence behavior, based on the TTM and various behavioral motivations. This comprehensive approach deepens the understanding of both the positive and negative factors influencing adherence, as well as their implementation pathways. It also facilitates the development of targeted intervention strategies to enhance adherence, ultimately maximizing the health benefits of exercise.

## Abbreviations

COREQ: Consolidated Criteria for Reporting Qualitative Research
TTM: Transtheoretical Model
